# Wind Turbine Diagnosis under Variable Speed Conditions Using a Single Sensor Based on the Synchrosqueezing Transform Method

**DOI:** 10.3390/s17051149

**Published:** 2017-05-18

**Authors:** Yanjie Guo, Xuefeng Chen, Shibin Wang, Ruobin Sun, Zhibin Zhao

**Affiliations:** School of Mechanical Engineering, Xi’an Jiaotong University, Xi’an 710049, China; guoyanjie@mail.xjtu.edu.cn (Y.G.); wangshibin2008@gmail.com (S.W.); sunruobin@stu.xjtu.edu.cn (R.S.); zhaozhibin@stu.xjtu.edu.cn (Z.Z.)

**Keywords:** wind turbine, vibration signal under variable speed condition, synchrosqueezing transform, local mean decomposition (LMD)

## Abstract

The gearbox is one of the key components in wind turbines. Gearbox fault signals are usually nonstationary and highly contaminated with noise. The presence of amplitude-modulated and frequency-modulated (AM-FM) characteristics compound the difficulty of precise fault diagnosis of wind turbines, therefore, it is crucial to develop an effective fault diagnosis method for such equipment. This paper presents an improved diagnosis method for wind turbines via the combination of synchrosqueezing transform and local mean decomposition. Compared to the conventional time-frequency analysis techniques, the improved method which is performed in non-real-time can effectively reduce the noise pollution of the signals and preserve the signal characteristics, and hence is suitable for the analysis of nonstationary signals with high noise. This method is further validated by simulated signals and practical vibration data measured from a 1.5 MW wind turbine. The results confirm that the proposed method can simultaneously control the noise and increase the accuracy of time-frequency representation.

## 1. Introduction

The global wind power capacity added 54 GW in 2016, bringing the total global installed capacity to nearly 487 GW [1,2]. With the increased generating capacity of wind turbines in the power grid, wind turbine fault diagnosis has become a significant issue which has a great impact on the operating conditions of wind turbines. When a wind turbine shuts down due to faults, great economic losses will occur [3,4,5,6]. The statistical analysis of global wind turbine failures, produced by the Caithness wind farm in 2016, shows that the number of failures is growing with the increased size of the wind market [7]. Wind turbines are complicated mechanical systems working in harsh environments, driven by natural wind which is changing over time, therefore, their key components are working under a high risk of failure. Due to the fluctuation of the rotation rate and the disturbance of strong background noise, the vibration signals, which carry the key information related to the health condition of rotating machinery, show strong nonlinear and non-stationary characteristics. When faults occur in the gears or bearings, the signals display amplitude-modulated and frequency-modulated (AM-FM) characteristics. Consequently, the diagnosis of gearbox vibration signals becomes more and more difficult [8,9]. As the maintenance devices for wind turbines are heavy and difficult to transport, the precise detection of incipient failures based on condition monitoring is important for the safety of wind turbines [10].

Plenty of techniques and theories have been developed focused on the fault detection of wind turbines. Tabatabaeipour et al. proposed a state space based set-membership fault detection method for fault detection of the benchmark model [11]. They also used the set-valued observers [12] to develop FDI methods for the uncertain linear time-varying systems of wind turbines. In Badihi, Zhang and Hong’s works [13,14,15], a number of algorithms were employed to design and develop integrated fault diagnosis and fault-tolerant control schemes for localizing sensor and actuator faults in wind turbines [16,17]. In addition, many scholars have used vibration sensors to measure the turbine acceleration signals, which are then processed in the frequency domain or time-frequency domain for the fault diagnosis of wind turbines.

Until now, numerous methods have been introduced to extract the fault-related signal components from the signals registered from wind turbines. The fast Fourier transform (FFT) is one of the most crucial techniques for efficiently analyzing the vibration signals. However, the FFT cannot get the characteristics between frequency and time [18,19,20]. Short-time Fourier transform (STFT) [21] and wavelet transform (WT) [22,23,24] can represent the time-frequency plane, but the time-frequency- representation (TFR) accuracy of these methods depends on the basis function selection. The changes of non-stationary signals are quite complex in practical engineering cases, which involves more characteristics of the inspected excitation. The Wigner-Ville distribution has a significant effect on the cross-terms [25], which affect the accurate interpretation of signals. Therefore, the STFT, WT, and WVT methods cannot be adapted to multi-component signals. Empirical mode decomposition (EMD) and local mean decomposition (LMD) are self-adaptive methods based on the characteristics of the signals [26,27,28,29]. The EMD and LMD decompose the original signals into different components, and provide the TFR simultaneously [30,31,32]. However, the analysis accuracy of the TFR is lower than that of the signal-scale frequency spectrum method because the multi-component signals, impacted by different forces with different amplitudes and different frequency response, are very complex. Synchrosqueezing transforms (SST) is a novel time-frequency (TF) post-processing method, which was proposed by Daubechies et al. in 2011 [33]. Compared to other TF representation enhancement methods, the SST method improves the time-frequency resolution and offers better adaptability and less deformation for instantaneous frequency (IF) profiles, and an exact reconstruction formula for constituent components [34,35]. SST can suppress the tiny noise of low frequency and slowly variable signals, but is still not suitable for processing signals with high noise levels.

In this paper, we propose a method to combine the LMD and SST for detecting nonstationary AM-FM signals with high noise levels. In Section 2, LMD and SST were analyzed by numerical simulations and an index is given to evaluate the accuracy of fault diagnosis. In Section 3, the proposed method is illustrated and the simulated signals with different SNRs are analyzed to validate the efficiency of the developed method. In Section 4, an experimental validation using data collected from a gearbox is presented. In addition, the proposed method is applied to test the drivetrain of a wind turbine. Conclusions are given in Section 5.

## 2. Theoretical Analysis

### 2.1. The Local Mean Decomposition Method

The vibration signals of gearboxes in wind turbines are non-stationary and highly noisy. Such signals contain the gear and bearing information and display AM-FM characteristics. In order to maintain the characteristics of the signals, LMD is the ideal denoising method.

The original multi-component signal can be decomposed into a series of frequency-modulated and envelope signals by the LMD method, similar to EMD. The product of each modulated signal and corresponding envelope signal is defined as a product function (PF).

The local extreme points of the original signal *x*(*t*) are denoted as *n_i_* (*i* = 1, 2, …). *i*-th means the value *m_i_* is the mean of two successive extreme *n_i_* and *n_i_*_+1_, given by *m_i_* = (*n_i_* + *n_i_*_+1_)/2. The *i*-th envelope estimate *a_i_* is given by *a_i_* = |*n_i_* − *n_i_*_+1_|/2. Given any signal *x*(*t*), it can be decomposed as follows:

(1) Initialize parameters. Set the index of PF *i* = 1, and residual signal *u*_0_ = *x*(*t*).
(2) Extract the *i*-th PF.
  (a) *h*_11_(*t*) = *u*_0_(*t*) − *m*_11_(*t*)
  (b) The local envelope estimates are smoothed in the same way as the local means to derive the envelope function *s*_11_(*t*) = *h*_11_(*t*)/*a*_11_(*t*)
  (c) Until |*s*_1*n*_(*t*)| ≤ 1, $a_{1}(t)=a_{11}(t)a_{12}(t)\ldots a_{1n}(t)=\prod_{q=1}^{n}a_{1q}(t)$, the calculation process of PF:
    (1) $${\mathrm{PF}}_{1}(t)\;=\;a_{1}(t)s_{1n}(t)$$
(3) PF is then subtracted from the original data *x*(*t*), resulting in a new signal *u*_1_(*t*) = *u*_0_(*t*) − PF_1_(*t*)
(4) Repeat the whole process *k* times until the *u_k_*(*t*) is constant or monotonic. Thus, we can achieve a series of decompositions and the final residue, explaining as follows:
  (2) $$x(t)=\sum_{p=1}^{k}{\mathrm{PF}}_{p}(t)+u_{k}(t)$$

The IF of PF*_i_* is calculated by:

(3) $$f_{i}(t)=\frac{d\arccos(s_{i}(t))}{2\pi dt}$$

The time-frequency distribution can be constructed based on the LMD results, namely displaying the instantaneous amplitude and instantaneous frequency of all PF components together.

The high-frequency mechanical vibration signals usually contain noise. The vibration signals of gearbox which is running in harsh environment are always contaminated by strong noise. The LMD can decompose such signals into several layers. The noise can influence the effective layers decomposition order [36]. In order to illustrate the signal with strong noise and investigate the robustness of LMD method to noise, a multi-component AM-FM signal *x*(*t*) is considered as follows:

(4) x(t)=cos(200πt+1.5sin(18πt))⋅(1+0.5cos(14πt))

**Figure 1:**
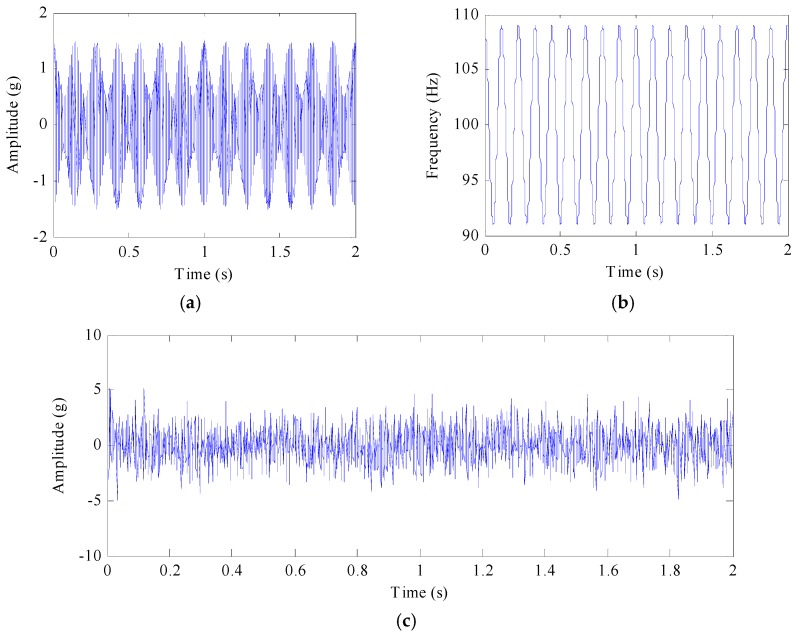
The simulated signal. (**a**) the signal *x*(*t*) = (1 + 0.5cos(14π*t*)) cos(200π*t* + 1.5sin(18π*t*)) for 0 < *t* < 2; (**b**) its IF, (**c**) the signal *z*(*t*) defined by *x*(*t*) with −5 dB noise.

A synthetic signal is the Gaussian noise *n*(*t*) added to *x*(*t*). The simulated signal and its spectrum are shown in Figure 1a,b. The signal whose SNR = −5 dB is shown in Figure 1c. The formula of the synthetic signal is written as:

(5) z(t)=x(t)+n(t)=cos(200πt+1.5sin(18πt))⋅(1+0.5cos(14πt))+n(t)

where *n*(*t*) is the noise function

**Figure 2:**
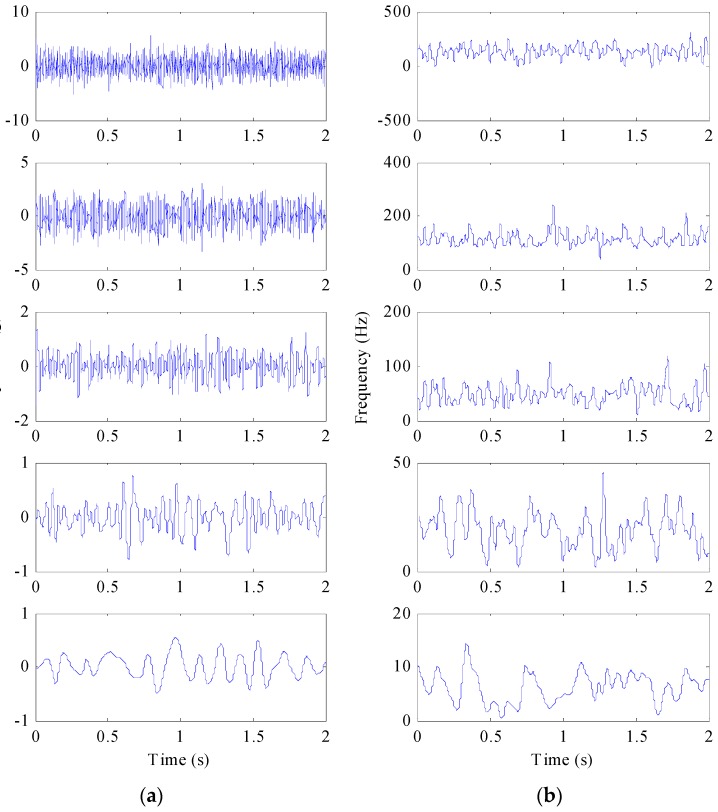
The decomposition of *x*(*t*) with SNR of −5 dB by LMD: (**a**) PFs in the time domain; (**b**) PFs in the frequency domain.

The signal is decomposed into five PFs as shown in Figure 2. Figure 2a gives the first five main components of the signal with the same time axis. Figure 2b is the frequency spectrum of the first five components, with different frequencies ranged from high to low. When the SNR = −5 dB, the first layer is similar to *x*(*t*). However, with the enhancement of noise, *x*(*t*) is decomposed gradually into the second and following layers. The reason for such a result can be interpreted as follows: Gaussian white noise signal contaminates all frequency parts of the signal. The signal with Gaussian white noise means that there is a high frequency components relative to the original signal. Therefore when such a signal is decomposed by LMD, white noise high-frequency components will be separated in PF_1_, which can greatly reduce the noise pollution in other PFs.

**Figure 3:**
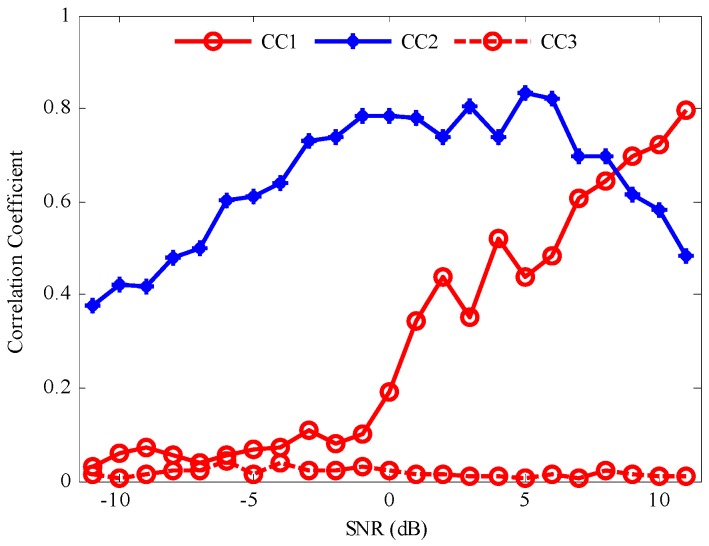
The correlation coefficients between *x*(*t*) and their corresponding PF components.

In order to study the effect of noise enhancement on signal decomposition, signals of different SNRs are decomposed and the corresponding correlation analysis is given. A total five PFs are obtained by LMD. For the purpose of studying correlations between PFs and *x*(*t*), only the first two PFs and the relevant IFs of *z*(*t*) are given. Then the correlation coefficient (CC) of the given PFs and *x*(*t*) is obtained, which denotes the relative amplitude and IF errors. Figure 3 shows the CCs of the given PFs and *x*(*t*) calculated at different SNRs. CC1 represents the correlation coefficient of *x*(*t*) and PF_1_. CC2 represents the correlation coefficient of *x*(*t*) and PF_2_. It can be observed that the CC1 decreases gradually with the increase of SNR. The small error value indicates that the effective components of *x*(*t*) have been separated out accurately. However, the changing trend is opposite to CC2, suggesting that the decomposition order is influenced by the noise level. The reason lies in the fact that decomposition order of LMD has been changed by the increasing noise. At high SNR level, the noise has less influence and the effective PFs are separated from signal firstly. With the decrease of SNR, the effective PFs are separated later and the decomposition errors increase gradually. When the SNR is below 8 dB, the high frequency noise is firstly separated from *z*(*t*) as a single PF. The above results indicate that the added high frequency noise changes the decomposition order and the effective PF components are separated at the second layer or later. However, the solution process of the IF by LMD is calculated by Equation (3), following the signal decomposition process. For the AM-FM signal, the IF of each component calculated by LMD is the derivative of FM signal, which ignores the impact of AM signal. Moreover, it is difficult to find the relevant components for FM signal by LMD.

### 2.2. Synchrosqueezing Transform

SST is a useful method to represent the time-frequency structure of signals. The steps of the SST are described as follows [33]:

First, the STFT $S_{x}(u,\xi )$ of the signal is calculated by the following formula. We use a purely harmonic signal $x(t)=Ae^{i2\pi f_{0}t}$. According to Parseval’s theorem, the STFT is given by:

(6) $$S_{x}(u,\xi )=\frac{1}{2\pi }\int_{-\infty }^{+\infty }\hat{x}(\omega )\bar{{\hat{g}}_{\sigma }(\omega -\xi )e^{-i\omega u}}d\omega =A{\hat{g}}_{\sigma }(2\pi f_{0}-\xi )e^{i}$$

where $g_{\sigma }(\omega )$ is the window function.

The STFT representation of the above harmonic signal x(t) will have its energy spread out in the TF plane around the line $\xi =2\pi f_{0}$.

Moreover, the time-shift derivative of signal’s TF representation $S_{x}(u,\xi )$ is given by:

(7) $$\partial_{u}S_{x}(u,\xi )=i2\pi f_{0}\cdot S_{x}(u,\xi )$$

which implies that a candidate IF $f_{x}(u,\xi )$ for the signal *x*(*t*) can be calculated approximately by the ratio of the time-shift derivative $\partial_{u}S_{x}(u,\xi )$ to the TF representation itself on the support of $S_{x}(u,\xi )$ as follows:

(8) $$f_{x}(u,\xi )=\{\begin{array}{ll} \left|\frac{\partial_{u}S_{x}(u,\xi )}{2\pi S_{x}(u,\xi )}\right|, & \left|S_{x}(u,\xi )\right|>\gamma \\ \infty , & \left|S_{x}(u,\xi )\right|\leq \gamma . \end{array}$$

where the parameter γ>0 is a hard-threshold on $\left|S_{x}(u,\xi )\right|$ to overcome the shortcoming that $\left|S_{x}(u,\xi )\right|\approx 0$ is rather unstable when signals have been contaminated by noise.

The final step of the SST is energy reassignment by summing different contributions:

(9) $$T_{x}(u,f_{l})=\sum_{\xi_{k}:\left|f_{x}(u,\xi_{k})-f_{l}\right|\leq \Delta f/2}S_{x}(u,\xi_{k})(\Delta \xi )$$

where the value $\xi_{k}$ is the discrete frequency with $\xi_{k}-\xi_{k-1}=\Delta \xi$, and the synchrosqueezing is determined only at the frequency centre $f_{l}$ of the interval $\left[f_{l}-\frac{1}{2}\Delta f,f_{l}+\frac{1}{2}\Delta f\right]$, with $\Delta f=f_{l}-f_{l-1}$. The STFT-based SST transforms the information from the (u,ξ) plane to another $\left(u,f_{x}(u,\xi )\right)$ plane.

**Figure 4:**
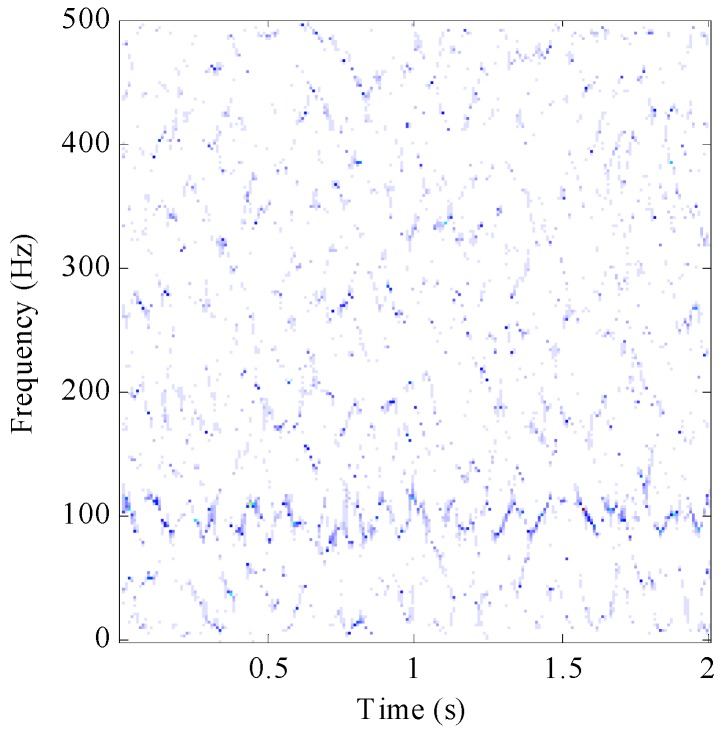
SST method result for simulation signal with −5 dB noise.

However, the SST method cannot separate features effectively when the signals contain strong background noise. The result of applying the SST method to represent the simulated signals (4) is shown in Figure 4. We can find that the IF is not clear in the TF figure as characteristics of the signal are masked by the high-intensity noise.

### 2.3. Characteristic Index

Since the signal contains speed information, a parameter was given to prove the effectiveness of the proposed method in denoising, according to the estimated speed in TF diagram and real speed [37].

$TF(t_{n},f_{m})$ (*n* = 1, 2, …, *N*; *m* = 1, 2, …, *M*) is the other TF representation ,where *N* and *M* are corresponding to the number of discrete time and discrete frequency in the TF representation, and $\left(t_{K},{\tilde{f}}_{K}^{C}\right)$ is the starting point in the TF plane.

Then, we define the cost function $C_{n,m}$ for the other N−1 time instants as:

(10) $$C_{n,m}=\{\begin{array}{ll} {\left|f_{m}-{\tilde{f}}_{n+1}^{C}\right|}^{2}-e_{n}{\left|TF(t_{n},f_{m})\right|}^{2}, & n=1,2,\ldots ,K-1\\ {\left|f_{m}-{\tilde{f}}_{n-1}^{C}\right|}^{2}-e_{n}{\left|TF(t_{n},f_{m})\right|}^{2}, & n=K+1,K+2,\ldots ,N \end{array}$$

where $f_{m}$ (m=1,2,…,M) are the candidate for seeking the ridge, and ${\tilde{f}}_{n}^{C}$ is local frequency at the time $t_{n}$ on the expectant IF curve which is defined as the frequency minimizing the cost function $C_{n,m}$:

(11) $${\tilde{f}}_{n}^{C}=\arg\underset{f_{m}}{\min}C_{n,m}=\{\begin{array}{ll} \arg\underset{f_{m}}{\min}{\left|f_{m}-{\tilde{f}}_{n+1}^{C}\right|}^{2}-e_{n}{\left|TF(t_{n},f_{m})\right|}^{2}, & n=1,2,\ldots ,K-1\\ \arg\underset{f_{m}}{\min}{\left|f_{m}-{\tilde{f}}_{n-1}^{C}\right|}^{2}-e_{n}{\left|TF(t_{n},f_{m})\right|}^{2} & n=K+1,K+2,\ldots ,N \end{array}$$

The parameter $e_{n}$ is changing with time used to adjust the proportion of the large amplitude and the smoothness of the IF curve in the cost function $C_{n,m}$ as:

(12) $$e_{n}={\left[\frac{f_{w}}{\underset{f_{m}\in FB_{n}}{\max}TF(t_{n},f_{m})}\right]}^{2}$$

where $f_{w}$ is the half-width of the frequency band.

$FB_{n}$ is the search range which is dynamically restricted to a small frequency band instead of the whole frequency range for ridge extraction, as:

(13) $$FB_{n}=\{\begin{array}{ll} \left\{f_{m}|{\tilde{f}}_{n+1}^{C}-f_{w}\leq f_{m}\leq {\tilde{f}}_{n+1}^{C}+f_{w},1\leq m\leq M\right\}, & \mathrm{for}\;n=1,2,\ldots ,K-1\\ \left\{f_{m}|{\tilde{f}}_{n-1}^{C}-f_{w}\leq f_{m}\leq {\tilde{f}}_{n-1}^{C}+f_{w},1\leq m\leq M\right\}, & \mathrm{for}\;n=K+1,K+2,\ldots ,N \end{array}$$

Thus, the proposed parameter mean square error (MSE) to illustrate the method is given as follows:

(14) $$\mathrm{MSE}=\frac{{\sum_{n}\left[f_{i}(n)-\tilde{IF_{j}}(n)\right]}^{2}}{{\sum_{n}\left[{\tilde{IF}}_{i}(n)\right]}^{2}}$$

where *f_i_*(*n*) (*i* = 1, …, *N*) represents the estimated speed in TF diagram, ${\tilde{IF}}_{i}(n)$ is the corresponding to real speed, and *N* is the number of discrete time periods.

## 3. Fault Diagnosis Method Based on SST and LMD

### 3.1. The Proposed Method to Improve SST

**Figure 5:**
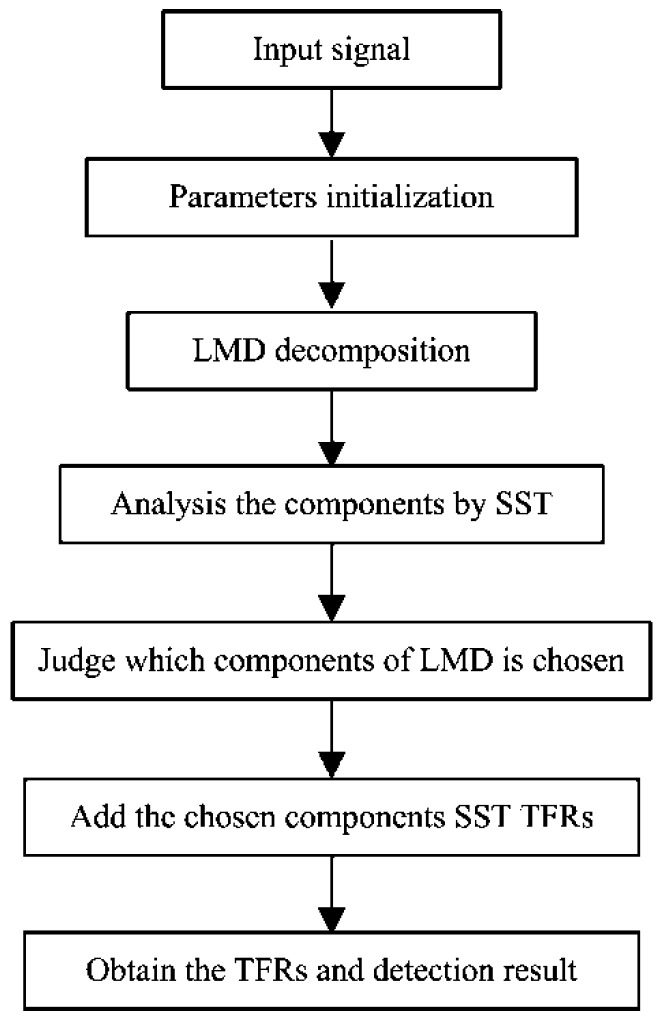
The processing flowchart of the proposed method.

As mentioned in Section 2, the LMD method is sensitive to noise and decomposes signals into several layers. When the SNR is low, it is possible that the signals in the first layer decomposed by LMD are Gaussian noise, which does not contain effective information. The middle layers contain the characteristics of the signal, including the fault information, and the last layer is residue. Therefore, the key issue for fault diagnosis of wind turbine is how to extract the amplitude information of the middle PFs, which can be solved by SST. Based on this issue, an improved method is proposed in this work. The basic principle is: firstly, the original signal is decomposed into different components by the LMD method, and the different components are PFs of the signal which contains the fault characteristics. Secondly, the first to the third PFs compressed by the SST method in TF representation are analysed, and the components with white noise are eliminated. Finally, the whole time-frequency structure is displayed by adding the TFR of the chosen components. Then, one can detect the fault clearly in TF representation. The flow chart of the improved SST method is shown in Figure 5. Here it should be mentioned that the proposed method is a not conducted in real time.

### 3.2. Signal Feature Analysis

**Figure 6:**
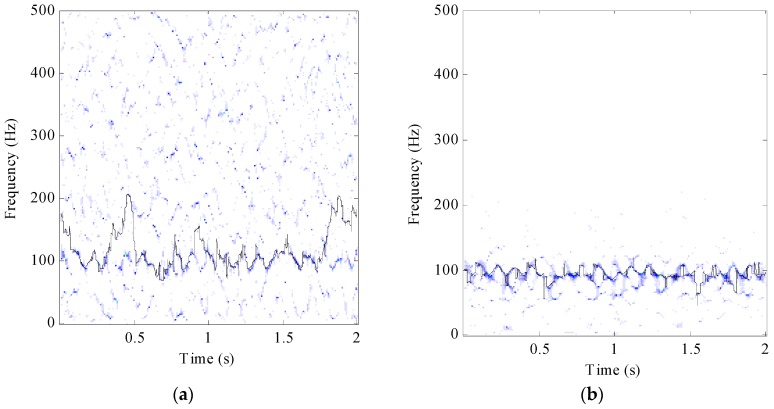
(**a**) TFR of simulated signal with −5 dB by SST; (**b**) TFR of simulated signal with −5 dB by the proposed method.

In order to evaluate the error of the SST and the proposed method, the IF *f*(*t*) is calculate from the TFR and the error rate is given by Equation (14). The simulated signal (4) with −5 dB was processed by SST as shown in Figure 6a and the MSE is 0.126. In comparison, the MSE decreases to 0.0126 when the same signal was processed by the proposed method, as shown in Figure 6b.

**Figure 7:**
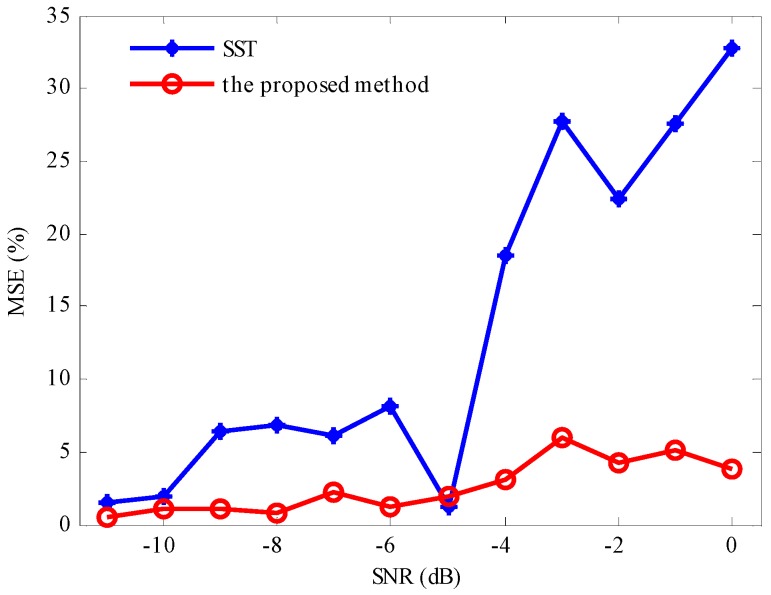
The comparison of error rate between SST and the proposed method.

Thereafter, Gaussian white noise with different SNRs level are added into the AM-FM signal *x*(*t*). The decomposition results by LMD showed that the PF_2_ had the highest correlation with the simulation signal as shown in Figure 3. Thus, one can compressed PF_2_ into time-frequency and evaluated the IF, when SNR is less than 0 dB. Compared with SST, it is found that the developed method performs better in denoising and accuracy in Figure 7.

## 4. Experiment Verification and Field Test

### 4.1. Experiment Verification

**Figure 8:**
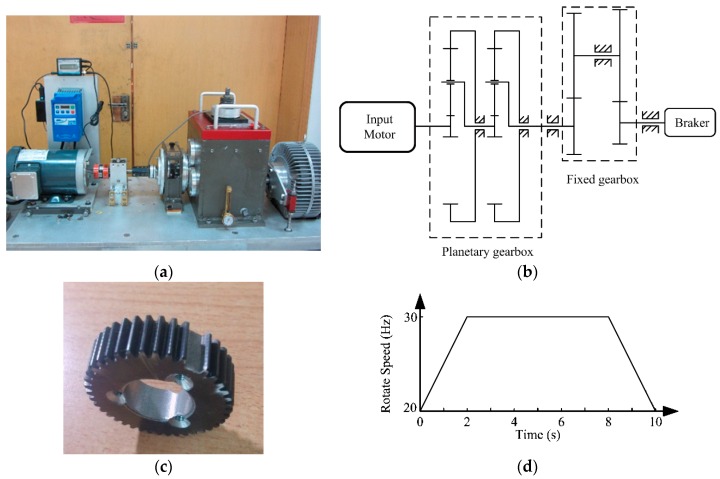
The planetary gearbox test rig: (**a**) Experimental system; (**b**) Schematic diagram of the rig; (**c**) Damage planetary gear missing tooth; (**d**) Input motor speed curve.

In order to demonstrate the effectiveness of the proposed method, a wind turbine driving chain fault simulation test rig is established and the corresponding verification experiments are conducted. The experimental system is presented in Figure 8a and the schematic diagram of the test rig is shown in Figure 8b.

In the verification, a missing tooth fault on the planetary gear in the first stage of planetary gearbox is created as shown in Figure 8c. For simulating wind turbine variable speed working conditions, the input motor speed was set up in advance to folow the speed curve shown in Figure 8d, changing from 20 Hz to 30 Hz. As shown in Figure 8b, there are two gearboxes in the test rig: a two-stage planetary gearbox and a two-stage fixed-axis gearbox. The present study just concerns the two-stage planetary gearbox. Table 1 lists the gear parameters. Since vibration-based analysis is one of the principal tools for diagnosing mechanical faults, the vibration signal is registered for the missing tooth using a single-axial accelerometer. The accelerometer (model number 356A12) is produced by PCB Electronics (La Terrasse, French). It is mounted on the planetary gearbox casing.

The fault feature frequency is calculated using the following formulas [38,39]:

(15) $$f_{m}=f_{c}Z_{r}=(f_{s}{}^{\left(r\right)}-f_{c})Z_{s}$$

(16) $$f_{P}=\frac{f_{m}}{Z_{P}}$$

where *f_m_* represents the mesh frequency; *f_c_* represents carrier rotating frequency; $f_{s}{}^{\left(r\right)}$ represents sun gear absolute rotating frequency; *f_P_* is fault feature frequency of the planetary gear; *Z_P_* is the number of planetary gears; and *Z_r_*, and *Z_s_* represents the number of teeth on ring gear and sun gear respectively. The calculated frequency of the gear box is given in Table 2.

**Figure 9:**
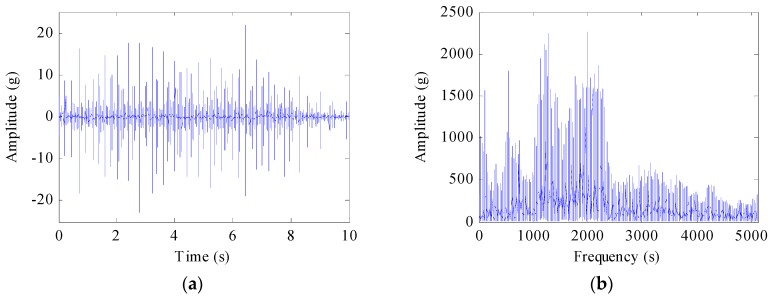

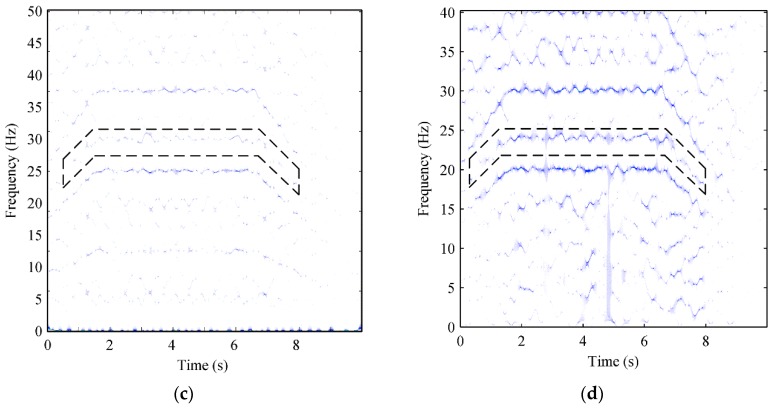
(**a**) Original vibration signal of the gearbox in time domain; (**b**) the spectrum of the vibration signal in frequency domain; (**c**) TFR of the signal using the SST method; (**d**) TFR of the signal using the proposed method.

Figure 9a,b present the tested vibration signal of gearbox with a missing tooth fault in time and frequency domain, respectively. One can see the noise of the original signal is low. This lies in the fact that the tests are conducted in lab. In order to verify the effectiveness of the proposed method under strong noise, Gaussian white noise SNR = 0 dB is added into the original vibration signal. Then the TF representations of the modified signal obtained by SST and the proposed method are shown in Figure 9c,d.

Compared with Figure 9c, the proposed method can capture the frequency changing from 18 Hz to 25 Hz, corresponding to the planetary gear fault frequency from Table 2. It verifies that the planetary gear of gearbox is faulty. Compared with the gear shown in Figure 9, one can find that there is a missing tooth in the gear. This test proves that the proposed method is effective.

### 4.2. Application to Fault Diagnosis of Wind Turbines

**Figure 10:**
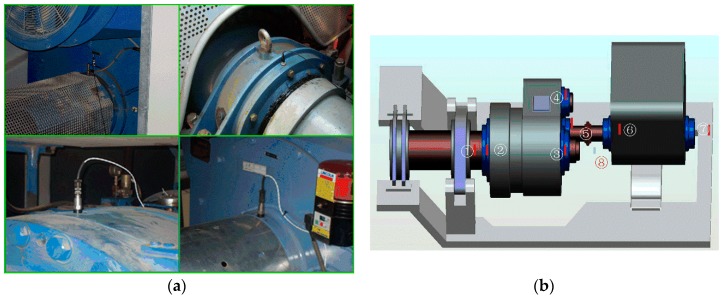
(**a**) Experimental setup; (**b**) Schematic of the sensor distribution on drivetrain of the wind turbine.

In order to validate the applicability of the presented method under actual working conditions, the vibration data generated from a wind turbine is taken for analysis to diagnose a faulty bearing in the gearbox. The experimental setup and sensor distributions on drivetrain of the wind turbine are shown in Figure 10. The wind turbine is a 1.5 MW double-fed induction generator with two gearboxes which contain a one-stage planetary gearbox and a two-stage fixed-axis gearbox. Figure 10a shows the sensor placement in the wind turbine and Figure 10b is the sensor distribution of the drivetrain of the wind turbine.

The detection system includes a personal computer, an offline data acquisition system, seven vibration acceleration transducers (①–⑦) and a proximity switch for rotational speed test (⑧). The rotational speed of the generator is variable and the sample frequency *f*_s_ is 25,600 Hz. The bearing used in this wind turbine is the deep groove ball bearing with the type of FAG6326C3 and the parameters changing with the speed of this bearing are presented in Table 3.

**Figure 11:**
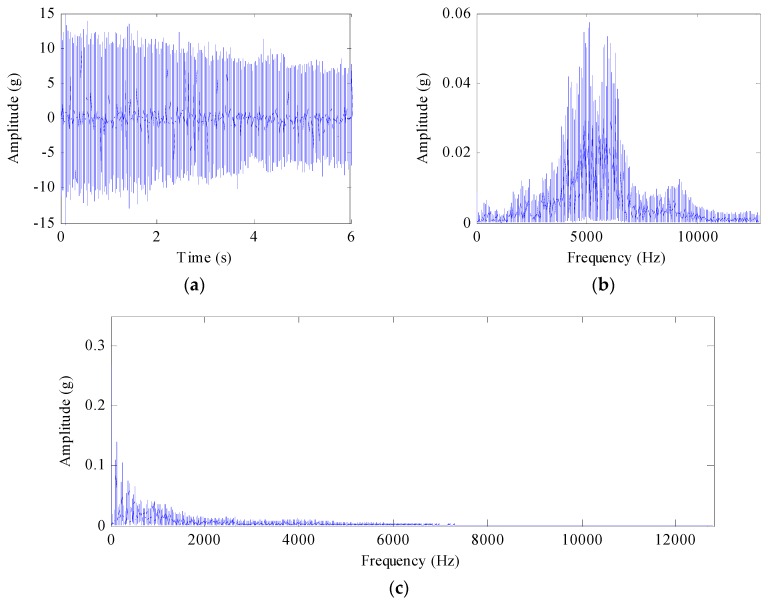
(**a**) Original vibration signal of the bearing in time domain; (**b**) the frequency of the vibration signal in frequency domain; (**c**) the envelope spectrum of the original signal.

The speed is changing from 26.7 Hz to 23.2 Hz. Figure 11a shows the original vibration signal of the bearing in the high speed stage of the gearbox (at measuring point 5). The amplitude decreases with the drop of rotating speed and load. Figure 11b gives the frequency of the vibration signal and it is difficult to find the fault frequency of the drivetrain. The peak group can be found in the envelope spectrum of the original signal as shown in Figure 11c, but one can hardly find the fault of the bearing because of the variable speed.

In order to find the fault precisely, we used the proposed method to analyze the original signal. However the mismatch between the high sampling frequency and the limited sampling frequency makes it difficult to deal directly with the signal by the time-frequency analysis method. Since the signal has low modulation frequency, we firstly envelope the original signal and drop the sampling frequency 64 times to improve the computation efficiency. Then the tested signal is processed by SST and the proposed method.

**Figure 12:**
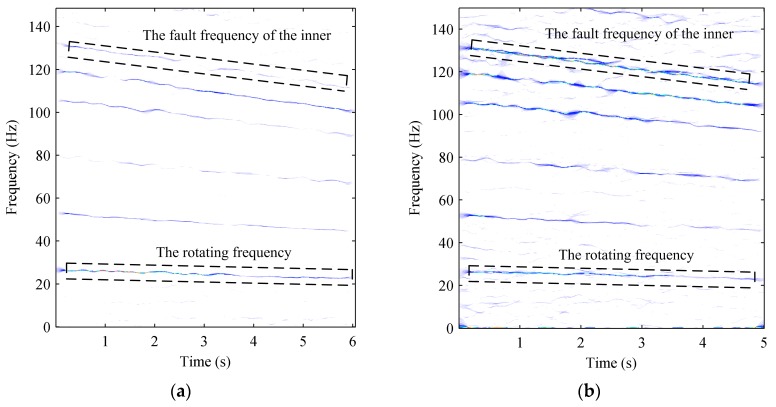
TFRs of the bearing signal: (**a**) TFR obtained by SST; (**b**) TFR obtained by the proposed method.

**Figure 13:**
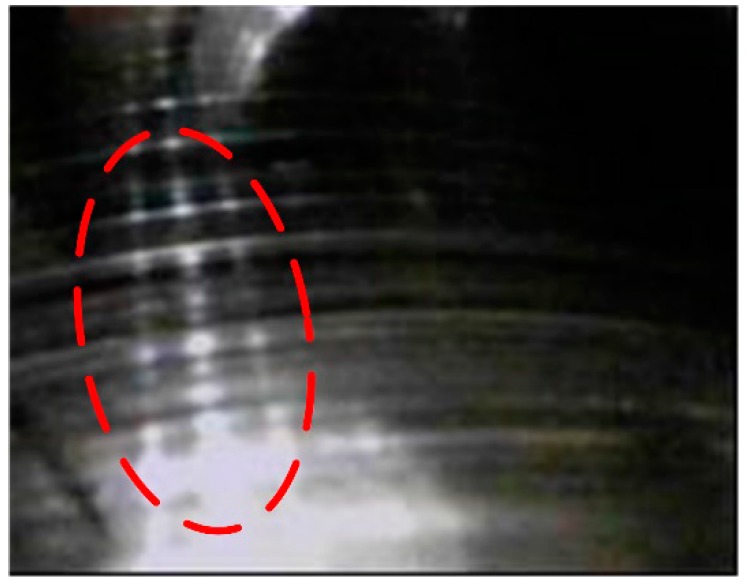
The inner ring of the bearing in the high stage of the gearbox.

Figure 12a gives the results processed by SST. As seen in the identification results, the fault feature cannot be detected for the experimental bearing because of the interference of the heavy background noise. Figure 12b illustrates the results processed by the proposed method. It is found that the rotation speed changes from 26.7 Hz to 23.2 Hz and the other TF line is changing from 130 Hz to 114 Hz, corresponding to the inner ring fault character frequency. This means that the inner ring of the bearing in the high stage of the gearbox is faulty. Comparing with the bearing shown in Figure 13, one can find that there are severe scratches on the surface of the inner ring. Therefore, this example gives powerful evidence of the superiority of the developed method for fault detection in high noise detection situations.

In this section, the proposed method has been applied to analyze the vibration signals carrying fault information which are taken from the experiment and practical application. The results verified the effectiveness of the proposed method in finding the characteristic fault frequencies of rotating machines under variable speed conditions with high noise.

## 5. Conclusions

In this paper, a novel method applied in non-real-time is proposed to improve SST for fault diagnosis of the variable speed signals in wind turbines using a sensor. In the proposed method, both local mean decomposition and synchrosqueezing transform were employed to decompose and compress the original signal with variable speed in the wind turbine. The signal is firstly decomposed into serial components by the LMD method, and then effective components are chosen and compressed by the synchrosqueezing transform. Thereafter, the compressed components are coupled and displayed. Through simulated analysis and experimental verification, it is found that the proposed method can denoise signals efficiently and improve the accuracy of TF resolution.

## Figures and Tables

**Table 1:** Gear parameters of the fixed gearbox.

	Sun	Planet	Ring
1st stage	20	**40 (3)**	100
2nd stage	28	36 (4)	100

**Table 2:** Characteristic frequency of the planetary gearbox.

	Meshing Frequency	Local Damage		
		Sun	Planet	Ring
1st stage	16.667 *f*_s_	2.5 *f*_s_	0.833 *f*_s_	0.5 *f*_s_
2nd stage	3.646 *f*_s_	0.521 *f*_s_	0.203 *f*_s_	0.146 *f*_s_

**Table 3:** The fault frequency of FAG6326C3.

Type	Frequency
The frequency of outer ring	3.133 *f*
The frequency of inner ring	4.867 *f*
The frequency of roller	2.197 *f*
The frequency of separator	0.392 *f*
